# Real-World Use of Isavuconazole as Primary Therapy for Invasive Fungal Infections in High-Risk Patients with Hematologic Malignancy or Stem Cell Transplant

**DOI:** 10.3390/jof8010074

**Published:** 2022-01-13

**Authors:** Hiba Dagher, Ray Hachem, Anne-Marie Chaftari, Ying Jiang, Shahnoor Ali, Rita Deeba, Shivan Shah, Issam Raad

**Affiliations:** Department of Infectious Diseases, The University of Texas MD Anderson Cancer Center, Houston, TX 77030, USA; HRDagher@mdanderson.org (H.D.); achaftari@mdanderson.org (A.-M.C.); yijiang@mdanderson.org (Y.J.); SMAli5@mdanderson.org (S.A.); REDeeba@mdanderson.org (R.D.); SShah11@mdanderson.org (S.S.); iraad@mdanderson.org (I.R.)

**Keywords:** isavuconazole, invasive aspergillosis, antifungal, anti-mold

## Abstract

(1) Introduction: Invasive fungal infections (IFIs) are a major cause of morbidity and mortality among immunocompromised patients with hematologic malignancies (HM) and stem cell transplants (SCT). Isavuconazole was approved by FDA as a primary therapy for Invasive Aspergillosis (IA) and Mucormycosis. The aim of this study is to look at the real-world use of Isavuconazole in patients with HM and evaluate their clinical outcomes and safety. (2) Methods: We conducted a retrospective study of HM patients at MD Anderson Cancer Center who had definite, probable or possible mold infections between 1 April 2016 and 31 January 2020 and were treated with Isavuconazole for a period of at least 7 days. Clinical and radiological findings were assessed at baseline and at 6 and 12 weeks of follow up. (3) Results: We included 200 HM patients with IFIs that were classified as definite (11), probable (63) and possible (126). Aspergillus spp was the most commonly isolated pathogen. The majority of patients (59%) received prophylaxis with anti-mold therapy and Isavuconazole was used as a primary therapy in 43% of patients, and as salvage therapy in 58%. The switch to Isavuconazole was driven by the failure of the primary therapy in 66% of the cases and by adverse effects in 29%. Isavuconazole was used as monotherapy in 30% of the cases and in combination in 70%. Adverse events possibly related to Isavuconazole were reported in eight patients (4%) leading to drug discontinuation. Moreover, a favorable response with Isavuconazole was observed in 40% at 6 weeks and in 60% at 12 weeks. There was no significant difference between isavuconazole monotherapy and combination therapy (*p* = 0.16 at 6 weeks and *p* = 0.06 at 12 weeks). Finally, there was no significant difference in outcome when Isavuconazole was used after failure of other anti-mold prophylaxis or treatment versus when used de novo as an anti-mold therapy (*p* = 0.68 at 6 weeks and *p* = 0.25 at 12 weeks). (4) Conclusions: Whether used as first-line therapy or after the failure of other azole and non-azole prophylaxis or therapies, isavuconazole seems to have a promising clinical response and a good safety profile as an antifungal therapy in high-risk cancer patients with hematologic malignancies. Moreover, combination therapy did not improve the outcome compared to Isavuconazole therapy.

## 1. Introduction

Despite advancements in antifungal therapy over the last two decades, invasive fungal infections (IFIs) continue to be a major cause of morbidity and mortality among immunocompromised patients, particularly if antifungal therapy is used inappropriately [1,2,3,4,5,6,7]. The newer triazole water-soluble pro-drug isavuconazonium sulfate (isavuconazole) demonstrated promising results in both in vitro and animal studies [8,9,10]. Isavuconazole has high bioavailability (98%), extensive tissue distribution, equivalent or higher fungicidal activity, and fewer drug-drug interactions, with no required age or renal dose adjustments [8,11,12,13]. In 2016, the SECURE trial demonstrated that isavuconazole had non-inferior efficacy and fewer drug-related adverse effects than voriconazole, specifically fewer hepatobiliary, eye, and skin toxicities; these results supported the use of isavuconazole as a safe and effective primary treatment for invasive aspergillosis (IA) [9].

The efficacy and safety of isavuconazole compared to that of amphotericin B was further demonstrated in the VITAL study, a single-arm open-label trial [14]. Since then, isavuconazole has been approved by the US Food and Drug Administration as a primary treatment for IA and mucormycosis mold infections [15]. It has also been approved by the European Medicines Agency for the same use when amphotericin B is not the best option [16]. The relatively recent approval of isavuconazole, combined with its safety and a broad spectrum of activity, has led to its increased and prolonged use, especially among hematologic malignancy patients at higher risk for mold infections [17], but only a few small studies have assessed its real world use among immunocompromised patients.

The current European Society of Clinical Microbiology and Infectious Diseases/European Confederation of Medical Mycology and ECIL-6 guidelines recommend isavuconazole or voriconazole as the first-line treatment for IA in hematologic malignancy patients [18,19], but voriconazole is still the first-line treatment according to the Infectious Disease Society of America guidelines [3].

Little or no information is available on the use of Isavuconazole in combination versus monotherapy or its use after anti-mold azole failure. The aim of this study was to evaluate the real-world use of isavuconazole in a large number of high-risk patients with hematologic malignancies and stem cell transplants and to evaluate these patients’ clinical characteristics and outcomes particularly in the setting of monotherapy versus combination therapy or if used after failure of other anti-mold prophylaxis as treatment versus as de novo treatment.

## 2. Materials and Methods

### 2.1. Patient Population

In this retrospective study, we screened the electronic medical records to identify all hematologic malignancy patients between the ages of 20 and 91 and who had received isavuconazole (either orally or intravenously) for at least 7 consecutive days 1 April 2016 and 31 January 2020 while inpatients at The University of Texas MD Anderson Cancer Center (Houston, TX, USA) and who had definite, probable, or possible mold infections, according to a list of isavuconazole administration cases obtained from the pharmacy analytics database [20]. Epidemiological and clinical data were collected using secure standardized forms and stored in our analytical file system, RedCap. This study was approved by the institutional review board at MD Anderson, and a patient waiver of informed consent was obtained.

### 2.2. Definitions

On the basis of the revised European Organization for Research and Treatment of cancer/mycosis Study Group definitions [20], a proven or definite mold infection was defined as documented histopathologic and microbiological evidence of mold infection in a tissue biopsy or needle aspiration specimen from a normally sterile site, excluding BAL, cranial sinus cavity, and urine. A probable mold infection was defined by the presence of at least one microbiological criterion (cytology, culture, or detection of antigen constituents with an aspergillus antigen test ≥1.0 optical density index unit from serum or BAL or ≥0.5 optical density index unit for two consecutive serum results), along with one host factor (recent absolute neutrophil count [ANC] < 500 cells/mL, allogeneic stem cell transplant, T-cell immune suppressant therapy, or prolonged corticosteroid use) and one clinical criterion (nodules, cavitary, or ground glass opacities found on computed tomography [CT]; tracheobronchitis; or sinonasal infection). A possible mold infection was defined as the presence of a host factor, along with a radiological criterion (nodules, cavitary, and ground glass opacities on CT).

The clinical and radiologic outcomes were evaluated at baseline and at 4 weeks, 6 weeks, and 12 weeks of follow-up. The baseline was defined as the start of isavuconazole therapy. Neutropenia was defined as an ANC < 500 cells/mL. Primary antifungal therapy was defined as the first therapy used upon diagnosis or suspicion of an IFI and administered for ≥7 days. Salvage antifungal therapy was defined as any therapy started after at least 7 consecutive days of no response to primary antifungal therapy. Breakthrough infection was defined as the development of a definite, probable, or possible IFI after at least 7 days of prophylaxis with an antifungal therapy with adequate anti-mold coverage. Patients on isavuconazole prophylaxis were excluded from this study. A favorable response included the complete or partial resolution of clinical, radiologic, and microbiologic findings. A complete response was defined as a complete resolution of clinical signs and symptoms, previously identified radiologic lesions on chest X-ray and CT, and all related microbiologic findings. A partial response was defined as a clinically significant improvement in signs and symptoms, improvement in radiologic abnormalities (≥50%), and no related microbiologic findings. Failure to respond included stability or progression of clinical or radiologic findings, persistent microbiologic findings, and death. IA-associated death was defined “as death after IA diagnosis in a patient with documented radiographic, microbiological or histological findings suggestive of active IA, ante-mortem or post-mortem, with no sustained favorable response to treatment” [21].

### 2.3. Statistical Analysis

This was a descriptive study, thus no power and sample size calculation were required or performed. Descriptive statistics were used to summarize patients’ data. Counts and percentages were reported for categorical variables, and medians and ranges were reported for continuous variables. Chi-square or Fisher’s exact test was used for the comparison of categorical variables between the two groups. Wilcoxon rank-sum test was used for the comparison of continuous variables. All the tests were two-sided, with a significance level of 0.05. The data analyses were performed using SAS version 9.3 (SAS Institute, Inc., Cary, NC, USA).

## 3. Results

### 3.1. Demographics and Clinical Characteristics

We screened 500 hematologic malignancy patients who had received isavuconazole for at least 7 consecutive days while inpatients at MD Anderson between 1 April 2016 and 31 January 2020; we identified 200 eligible patients who had been diagnosed with definite (11 patients), probable (63 patients), or possible (126 patients) IFIs. Among these 200 patients, 65% (129) were male, and their median age was 63 years (20–91 years). More than half (55%) had acute myeloid leukemia (AML), with ALL and CML being the second and third most common cancer diagnoses, respectively (Table 1). Fifty-two (26%) patients had undergone a stem cell transplant in the year prior to or during their IFI, with only two auto transplants and 50 allogeneic stem cell transplants. Furthermore, 26 patients had been diagnosed with graft-versus-host disease before or during the infection. In addition, 61% (122) had neutropenia, defined as ANC ≤ 500 cells/mL at the time of diagnosis or admission, with more than half (39%) recovering from neutropenia ANC > 500 cells/mL during the course of their IFI. Several host risk factors for developing an IFI included the use of steroids during the IFI in 82% (164) of patients, with 28% using a cumulative dose of ≥600 mg (prednisone equivalent). During the course of the infection, 86 patients (43%) were admitted to the intensive care unit and 50 patients required mechanical ventilation (25%). Eighty-one patients had a pulmonary co-infection (41%) in addition to their IFI.

### 3.2. Invasive Fungal Infection

Eleven patients (6%) were diagnosed with proven IFIs, 63 (32%) with probable IFIs, and 126 (63%) with possible IFIs (Table 1), with the majority involving invasive pulmonary infections (86%). Table 1 shows the types of IFIs identified. Most of these cases were IA (55 cases [74%]), with only 23 *Aspergillus* species isolated, while the rest were identified as part of the GM criteria mentioned above. There were 8 *Mucor* cases (11%). The median duration of isavuconazole use, with a maximum cut-off of 12 weeks marking the end of therapy, was 48 days (range, 21–97 days). Only 30% (59) of patients received isavuconazole alone, while 71% received it in combination with other antifungals, most commonly a polyene (54%) or echinocandin (27%).

Anti-mold prophylaxis was used in 112 (59%) patients for at least 7 consecutive days prior to the onset of infection, which means that 59% of our cases were breakthrough infections. The anti-mold agents were voriconazole, posaconazole, and echinocandins. Ninety patients received voriconazole or posaconazole prophylaxis, and 28 patients received echinocandin prophylaxis. For 10 patients, we could not find definitive information on whether they were or were not on prophylaxis.

Eighty-five patients used isavuconazole as primary therapy, 30 as monotherapy and 55 as combination therapy. Table 2(A) shows that the monotherapy group and the combination therapy group had comparable characteristics, with no significant differences except the type of cancer. The combination therapy group had a significantly higher percentage of AML cases: 53% compared to 23% in the monotherapy group (*p* = 0.009).

In the monotherapy group, 14 of 30 patients (47%) had a favorable response, while 16 (53%) did not respond at 6 weeks. On the other hand, in the combination therapy group, 18 of 55 patients (33%) showed a favorable response, while 37 (67%) did not respond. While the combination therapy group had a higher rate of failure to respond at 6 weeks (67%) and at 12 weeks (71%), these differences were not significant (*p* = 0.20 and *p* = 0.19, respectively). There were also no significant differences in the all-cause mortality rates at 6 and 12 weeks between the monotherapy and combination therapy groups (*p* = 0.19 and *p* = 0.12, respectively) nor the IFI-attributable mortality rates (*p* = 0.17 and *p* = 0.28, respectively) (Table 2(A)). Furthermore, Table 2(A) shows that when stratifying IFIs into definite/probable vs possible IFIs, in both the monotherapy and combination groups, the rates of favorable response are also similar at 6 and 12 weeks with no significant differences. Among the definite and probable cases, the favorable response at 6 and 12 weeks was 63% in the monotherapy group. There was only 1/8 all-cause mortality death in the monotherapy group for definite and probable IFIs, and 11 deaths in the combination therapy group.

### 3.3. Adverse Events

Adverse events that were possibly related to isavuconazole and led to drug discontinuation were reported in only nine patients (5%). These included five cases of elevated transaminases, which was defined as a three-fold increase in alanine aminotransferase (ALT), aspartate aminotransferase (AST), or bilirubin compared to the baseline. A three-fold increase was noted in ALT levels in two cases, AST levels in two cases, and both AST and ALT levels in one case. The other four adverse events (one case each) were nausea, profound fatigue, a hypersensitivity reaction, and hallucinations (Table 1).

### 3.4. Outcomes

As previously defined, the response to isavuconazole therapy was assessed on the basis of clinical, radiologic, and microbiologic findings at 6 and 12 weeks compared to the baseline (initiation of isavuconazole). At 6 weeks, 79 (40%) patients had a favorable response, and 121 (60%) did not respond, with an all-cause mortality rate of 25% (49 patients) and an IFI-attributable mortality rate of 21% (41 patients) (Table 1). At 12 weeks, 65 (33%) patients had a favorable response and 135 (67%) did not respond, with an all-cause mortality rate of 46% (92 patients) and IFI-attributable mortality rate of 37% (74 patients) (Table 1).

Isavuconazole was used as primary monotherapy in 30 cases and salvage monotherapy in 29 cases. In the monotherapy group, 28 patients (47%) had a favorable response at 6 weeks compared to 31 patients (53%) who did not respond. At 12 weeks, 25 patients (42%) had a favorable response compared to 34 patients (58%) who did not respond.

### 3.5. Primary and Salvage Outcomes

Isavuconazole was used as primary therapy in 85 cases (43%) and as salvage therapy in 115 cases (58%) (after at least 7 consecutive days of primary therapy with an anti-mold agent). The switch to isavuconazole as salvage therapy was driven by the failure of primary therapy in 57% of cases, subtherapeutic Posaconazole or voriconazole levels in 11%, and adverse effects in 29%. These adverse effects included elevated liver function tests (16%), a prolonged QT interval (8%), altered mental status (2%), hallucinations due to voriconazole (3%), and worsening creatinine (3%) (Table 1). There was only one case of nausea and vomiting and one of clinical study incompatibility; both cases represented 1% of the cases that involved switching to isavuconazole. Approved insurance coverage was the reason for 1% of switches; in 5%, the reason was not specified. In 50% of cases, voriconazole or Posaconazole was used as primary therapy before isavuconazole was used as salvage therapy. Other primary therapies before isavuconazole were polyene or an echinocandin in 47% and 27% of cases, respectively, whether used alone or in combination with other azoles. Isavuconazole was mostly used as salvage therapy (58% compared to 43% as primary therapy). In three cases, it was used as both primary and salvage therapy following the failure of other salvage therapies during infection. Polyenes and echinocandins were used alone or in combination as salvage therapies in 30% and 23% of cases, respectively. Only 26% of patients did not receive any salvage therapy (Table 1).

Of the 109 patients who switched to isavuconazole as salvage therapy because primary therapy failed or resulted in adverse effects, 27 were treated with isavuconazole monotherapy vs 82 patients treated with combination therapy. The two groups were comparable, with no significant difference in their clinical characteristics (Table 2(B)). At 6 weeks, 52% of the monotherapy group had had a favorable response, while 48% had not responded. In the combination therapy group, 37% of patients had a favorable response at 6 weeks and 63% had not responded, a non-significantly higher rate of failure to respond than that for isavuconazole monotherapy (*p* = 0.16). At 12 weeks, the favorable response rate was non-significantly higher in the monotherapy group than in the combination therapy group (44% vs 24%; *p* = 0.06) (Table 2(B)). In the monotherapy group, the favorable response was higher among possible IFIs as compared to definite and probable IFIs at 6 and 12 weeks (63% and 56% respectively) while it was similar between the two IFI groups that received combination therapy There was no significant difference in the mortality rates.

Table 3 compares the patients who did not respond to treatment with voriconazole or posaconazole (azoles) before switching to isavuconazole (group 1) with the patients who received isavuconazole without prior treatment with an azole (group 2); isavuconazole was given as primary treatment, with no prior anti-mold prophylaxis (de novo), or after the failure of a non-azole anti-mold therapy, namely echinocandins. The two groups, shown in Table 3, were comparable, with no significant differences in the majority of patient characteristics, with the exception of allo-bone marrow transplant and primary versus salvage therapy. A significantly higher percentage of patients in group 2 had a history of allo-bone marrow transplant than did those in group 2 (29% versus 13%, respectively; *p* = 0.02). As expected, the majority of patients in group 1 received isavuconazole as salvage therapy (74%), while the majority of patients in group 2 received isavuconazole as primary therapy (92%) (*p* < 0.0001). Group 1 had a favorable response of 38% at 6 weeks and 29% at 12 weeks. These results did not significantly differ from those in group 2, with a favorable response of 42% at 6 weeks and 38% at 12 weeks. The mortality rates were also very similar between the two groups at both 6 and 12 weeks. Stratifying both groups into definite/probable IFIs versus possible IFIs yielded similar favorable response rates and mortality rates in both the combination and the monotherapy groups at 6 and 12 weeks. (Table 3).

### 3.6. Neutropenia and Recovery

We identified 122 patients (61%) who had neutropenia (ANC < 500 cells/mL) at the onset of their IFI; 48 (39%) recovered during the IFI. As expected, patients with unresolved neutropenia had a significantly lower favorable response rate (9 of 74 [12%]) than did those with resolved neutropenia (27 of 48 [56%]; *p* < 0.0001). At 12 weeks, similar findings were observed: 44% of patients with neutropenia recovery had a favorable response, as opposed to 5% in the no neutropenia recovery group. Conversely, 56% of patients did not respond, despite neutropenia recovery, as opposed to 93% in the no neutropenia recovery group (*p* < 0.0001).

## 4. Discussion

To our knowledge, this is the first study of a large number of patients (200 patients) to evaluate the real-world experience with using isavuconazole as an anti-mold therapy. Our findings showed that isavuconazole monotherapy had similar outcomes compared to isavuconazole in combination therapy. We also showed that even when used after the failure of non-azole therapy or other azole therapies, such as posaconazole and voriconazole, or when used de novo, as an anti-mold therapy, isavuconazole still had a favorable outcome.

In the SECURE trial, Isavuconazole, used as a de novo antifungal, showed non-inferior efficacy compared to voriconazole, with all-cause mortality rates of 19% at 6 weeks and 29% at 12 weeks [9]. In our study, among the 59 patients who received isavuconazole as monotherapy, the all-cause mortality rate was 19% at 6 weeks, with an IFI-attributable mortality rate of 14%. At 12 weeks, the all-cause mortality rate was 36%, with an IFI-attributable mortality rate of 28%. A favorable response was seen in 47% of patients at 6 weeks and 42% at 12 weeks compared to the 35% reported in the SECURE trial.

In the VITAL trial, which included only patients with mucormycosis and compared isavuconazole to amphotericin B, patients who were given isavuconazole as primary therapy had a favorable response rate of 32% versus 36% when it was used as salvage therapy. The all-cause mortality rates were 38% at 6 weeks and 43% at 12 weeks [14]. We combined the outcomes of all of our patients, whether they used isavuconazole as monotherapy or as combination therapy and found that 40% had a favorable outcome at 6 weeks and 33% had a favorable outcome at 12 weeks, with all-cause mortality rates of 25% at 6 weeks and 46% at 12 weeks. These outcomes (in both the SECURE and VITAL studies) are also comparable to those seen among the subset of patients who switched to isavuconazole as salvage therapy because of the failure of anti-mold primary therapy or its adverse effects. Moreover, this specific subset of patients was not previously assessed in the SECURE trial, with a 6-week positive response rate of 39%, a 12-week favorable response of 30%, and an all-cause mortality rate of 27% at 6 weeks and 51% at 12 weeks.

Furthermore, Table 3 shows that even after the failure of other azole therapies, such as voriconazole and posaconazole, when switched to isavuconazole, patients had a favorable response of 38% at 6 weeks and 29% at 12 weeks, which is comparable to the results found in both the SECURE and VITAL trials. Similarly, the all-cause mortality rates were 24% at 6 weeks and 49% at 12 weeks. Even more so, when stratifying the monotherapy and combination therapy groups into definite/probable versus possible IFIs, similar results further confirmed the efficacy of Isavuconazole among all IFIs. Isavuconazole also showed comparable results when used as primary therapy, with no prior anti-mold treatment or prophylaxis, or when used after treatment with a non-azole therapy, such as echinocandin. To our knowledge, our study is the first to specifically show that switching to isavuconazole after other anti-mold therapies have failed did not affect the efficacy of or favorable response to isavuconazole.

Most of our patients (71%) received isavuconazole in combination with other anti-mold treatments, namely polyenes (54%) or echinocandins (27%). At 6 weeks and at 12 weeks, there was no significant difference between those who received isavuconazole in combination with other anti-mold therapies as primary therapy or salvage therapy and those who received isavuconazole as monotherapy. In comparison, in a randomized controlled trial of 454 patients with hematologic malignancy or HCT and suspected or documented IA, Marr et al. showed that overall mortality rates at 6 weeks for patients with IA who were treated with a combination of voriconazole and anidulafungin were not significantly different from those who were treated with voriconazole monotherapy (19.3% in the combination group vs. 27.5% in the monotherapy group; *p* = 0.087) [22]. In another retrospective study of 181 hematologic malignancy patients with IA, voriconazole and caspofungin combination therapy was not associated with improved outcomes compared to voriconazole monotherapy [21]. One observation from Table 2 is that combination therapy was initiated more often than isavuconazole monotherapy among patients with a worse clinical baseline.

One of the common risk factors for the development of IA in hematologic malignancy patients is neutropenia [3]. Neutropenia has long been associated with a less favorable response to antifungal therapy among hematologic malignancy patients [3,22,23]. In a post hoc analysis of data from the SECURE trial, which included 142 neutropenic patients with IA, isavuconazole was found to be effective and safe compared to voriconazole. In both groups, the resolution of neutropenia was associated with a lower all-cause mortality outcome [24]. As expected, the resolution of neutropenia in our study was associated with improved all-cause mortality and favorable outcomes at each timepoint.

Some of the toxicities associated with the use of triazoles include hepatotoxicity, peripheral neuropathy, skin rashes, visual hallucinations, and heart failure [17,25,26]. Adverse effects related to voriconazole specifically include visual, hepatic, dermatologic, and neurotoxic side effects [9,27,28]. Several studies have shown that isavuconazole has high tolerability and a good safety profile [9,17,29,30,31]. The SECURE trial demonstrated lower rates of hepatobiliary disorders (9%), eye disorders (15%), and skin disorders (33%) in patients who received isavuconazole than in those who received voriconazole [9]. A recent retrospective study among patients with chronic pulmonary aspergillosis treated for a prolonged period of 2 to 11 months showed significantly fewer adverse effects among those treated with Isavuconazole compared to voriconazole [32]. In a retrospective study of 50 hematologic malignancy patients, DiPippo et al. showed a lack of toxicity with isavuconazole despite long-term use, with only two reported cases of transaminitis, two paresthesias, and one alopecia [17]. In our study, we reported a total of nine adverse events (5%) that were possibly related to isavuconazole that led to drug discontinuation (five elevated transaminases, one nausea, one hypersensitivity reaction, and one hallucination).

The reported safety profile of isavuconazole compared to other triazoles, in combination with the prevalence of hepatobiliary disorders among hematologic malignancy patients, played a role in the switch to isavuconazole therapy in our patient population. One of the limitations of the SECURE trial was the exclusion of patients with hepatobiliary disorders and those receiving anti-mold prophylaxis [9]. With no such exclusion in this study, we were able to assess why clinicians switched to isavuconazole after a different anti-mold primary therapy in 58% of our patients. The switches were mainly driven by the failure of the primary therapy (54%); However, 29% of cases were caused by adverse effects related to primary therapy, including elevated liver function tests (16%), a prolonged QT interval (6%), altered mental status (2%), and decreased creatinine (3%). In a study of 30 patients, isavuconazole was also indicated because of adverse events associated with prior anti-mold therapy; the most common adverse event was hepatotoxicity, followed by renal insufficiency and long QTC intervals, according to Kronig et al. [33]. The study also showed that after switching to isavuconazole, liver function tests and QTc intervals decreased compared to the baseline recorded with prior azoles used (34). Thus, our data support the safety profile of isavuconazole and identify it as one of the main reasons to switch among hematologic malignancy and stem cell transplant patients.

The limitations of this study include its retrospective nature, being limited to one cancer center, and having more possible IFI cases than definite and probable IFI cases. Another limitation is that all patients were only assessed at 6 weeks and 12 weeks; it did not always account for clinical response at the end of isavuconazole therapy. Furthermore, although the outcome findings between monotherapy versus combination therapy were only a part of our exploratory analyses with statistical power limitation, we think they are worthy of note and may deserve a study in the future with a bigger sample size for a further investigation.

The strength of our study is the inclusion of a large cohort of hematologic malignancy patients with definite, probable, or possible IFIs and the inclusion of patients with breakthrough anti-mold prophylaxis and hepatobiliary disorders, something that had not been reported previously.

## 5. Conclusions

Whether used as first-line of therapy or after the failure of other azole and non-azole prophylaxis or therapies, isavuconazole seems to have a promising clinical response and a good safety profile as an antifungal therapy in high-risk cancer patients with hematologic malignancies. Overall, combination therapy did not improve the outcome compared to Isavuconazole therapy and despite the small sample size of this comparison, it is worth noting for further investigations. Finally, the selection of isavuconazole therapy among hematologic malignancy patients was mainly driven by the failure of other antifungal agents, subtherapeutic posaconazole or voriconazole levels, or adverse events with other antifungals, such as increased liver function tests and prolonged QT intervals.

## Figures and Tables

**Table 1:** Patient Characteristics and Outcomes.

Patients’ Characteristics	Patients, *n* (%) (*n* = 200)
Age (years), median (range)	63 (20–91)
Sex, male	129 (65)
Use of isavuconazole	
Primary therapy	85 (43)
Salvage therapy	115 (58)
Reason to switch to isavuconazole as salvage therapy	
Failure of primary therapy	66/115 (57)
Insurance coverage	4/115 (3)
Adverse effect of primary therapy	33/115 (29)
Elevated liver function tests	18/115 (16)
Prolonged QT	9/115 (8)
Altered mental status	2/115 (2)
Hallucinations from vori	3/115 (3)
Nausea/vomiting	1/115 (1)
Worsening creatinine	3/115 (3)
Other	21/115 (18)
Subtherapeutic posaconazole/voriconazole levels	13/115 (11)
Study incompatibility	1/115 (1)
Insurance approval	1/115 (1)
Non-specified	6/115 (5)
Duration of isavuconazole (days), median (IQR)	48 (21–97)
Isavuconazole used	
Alone	59 (30)
In combination	141 (71)
Isavuconazole used in combination with	
Polyene	107 (54)
Echinocandins	53 (27)
Terbinafine	1 (1)
Type of IFI ^1^	
*Aspergillosis*	55/74 (74)
*Mucor*	8/74 (11)
*Fusarium*	1/74 (1)
Others	10/74 (14)
Diagnosis of IFI	
Definite	11 (6)
Probable	63 (32)
Possible	126 (63)
Invasive pulmonary infection or sinus infection	177 (89)
Type of cancer	
AML	110 (55)
ALL	17 (9)
CLL	6 (3)
CML	10 (5)
Lymphoma	8 (4)
Myeloma	8 (4)
Other	41 (21)
BMT prior to or during IFI	52 (26)
Type of BMT	
Autologous stem cell transplant	2/52 (4)
Allogeneic stem cell transplant	50/52 (96)
Type of Allo BMT	
HLA-related ^2^	23/50 (46)
Matched unrelated	22/50 (44)
Umbilical cord	2/50 (4)
Others	3/50 (6)
GVHD	26/51 (51)
Neutropenia at the onset of IFI	122/199 (61)
Recovery from neutropenia (ANC > 500) during infection	48/122 (39)
Steroid treatment during infection	165 (83)
Cumulative dose of steroids received during infection	
≥600 mg (prednisone equivalent)	55/164 (34)
<600 mg (prednisone equivalent)	109/164 (66)
ICU during infection	86 (43)
Mechanical ventilation during infection	50/198 (25)
Positive fungal culture	47 (24)
Species identified	
*Aspergillus* spp.	23/47 (49)
*Candida* spp.	7/47 (15)
*Rhizopus* spp.	6/47 (13)
*Fusarium* spp.	1/47 (2)
Other species	10/47 (21)
Co-infection	142 (71)
Organism for co-infections	
Pulmonary co-infection	81 (41)
Type of co-infection	
Bacterial ^3^	107 (54)
Viral	52 (26)
Antifungal prophylaxis	129/190 (68)
Anti-mold prophylaxis	112/190 (59)
Type of prophylaxis	
Voriconazole and/or posaconazole	90/190 (47)
Echinocandins	28/190 (15)
Breakthrough	112/190 (59)
Primary therapy	
Polyene	94 (47)
Voriconazole and/or posaconazole	100 (50)
Isavuconazole	85 (43)
Echinocandins	54 (27)
Salvage therapy	
No salvage therapy	52 (26)
Polyene	59 (30)
Voriconazole and/or posaconazole	47 (24)
Isavuconazole	118 (59)
Echinocandins	45 (23)
**Response to Treatment**	
Response to isavuconazole therapy at week 6	
Favorable response	79 (40)
Unfavorable response	121 (60)
Complete response	12 (6)
Partial response	67 (34)
Failure to respond	114 (57)
Stable disease	7 (3)
Response to isavuconazole therapy at week 12	
Favorable response	65 (33)
Unfavorable response	135 (67)
Complete response	26 (13)
Partial response	39 (20)
Failure to respond	130 (65)
Stable disease	5 (2)
Adverse events related to isavuconazole leading to drug modification	9 (5)
Nausea	1
Profound fatigue	1
Hypersensitivity reaction	1
Hallucinations	1
≥× AST	2
≥×3 ALT	2
≥×3 ALT and AST	1
Adverse events resolved after drug modification	5/7 (71)
Death within 6 weeks of IFI diagnosis	49 (25)
IFI-attributable death within 6 weeks	41 (21)
Death within 12 weeks of IFI diagnosis	92 (46)
IFI-attributable death within 12 weeks	74/199 (37)

Abbreviations: IQR: interquartile, AML: Acute myeloid leukemia; ALL: Acute lymphocytic leukemia; CLL: Chronic lymphocytic leukemia; CML: Chronic myelogenous leukemia. GVHD: Graft-versus-host disease. BMT: Bone marrow transplant; ICU: Intensive care unit; ANC: Absolute neutrophil count. Notes: ^1^ Possible IFI excluded from types of IFI classification. ^2^ HLA-related: “Human leukocyte antigen”-related. ^3^ Bacterial Infection was defined as a positive bacterial culture.

**Table 2:** Treatment Outcomes.

(A) Patients with Isavuconazole as Primary Therapy			
Variable	Monotherapy, *n* (%) (*n* = 30)	Combination Therapy, *n* (%) (*n* = 55)	*p*-Value
Age (years), median (range)	64 (37–91)	63 (20–90)	0.33
Sex, male	21 (70)	34 (62)	0.45
Type of IFI (definite or probable)			
*Aspergillosis*	6 (20)	17 (31)	0.28
*Mucor*	0 (0)	3 (5)	0.55
*Fusarium*	0	0	
Diagnosis of IFI			0.56
Definite	1 (3)	3 (5)	
Probable	7 (23)	19 (35)	
Possible	22 (73)	33 (60)	
Invasive pulmonary infection or sinus infection	27 (90)	52 (95)	0.66
Type of cancer			0.009
AML	7 (23)	29 (53)	
Others	23 (77)	26 (47)	
BMT-Allo	5 (17)	13 (24)	0.45
GVHD	3 (10)	8/54 (15)	0.74
Neutropenia at the onset of IFI	14 (47)	35/54 (65)	0.11
Recovery from neutropenia during infection	7/14 (50)	11/35 (31)	0.22
Prophylaxis	16 (53)	38/52 (73)	0.08
Type of prophylaxis			
Voriconazole	3 (10)	5/52 (10)	>0.99
Posaconazole	7 (23)	21/52 (40)	0.12
Isavuconazole	0 (0)	1/52 (2)	>0.99
Echinocandins	5 (17)	9/52 (17)	0.94
Other	1 (3)	3/52 (6)	>0.99
Anti-mold prophylaxis	15 (50)	34/52 (65)	0.17
Cumulative steroids ≥ 600 mg (prednisone equivalent) during infection	9/29 (31)	11 (20)	0.26
ICU during infection	10 (33)	24 (44)	0.35
Mechanical ventilation during infection	6 (20)	15/54 (28)	0.43
Positive fungal culture	5 (17)	15 (27)	0.27
Species identified			0.81
*Aspergillus* spp.	3/5 (60)	6/15 (40)	
*Candida* spp.	0/5 (0)	3/15 (20)	
Others	2/5 (40)	6/15 (40)	
Co-infection	21 (70)	45 (82)	0.21
Bacterial co-infection ^1^	15 (50)	37 (67)	0.12
Viral co-infection	5 (17)	18 (33)	0.11
Favorable response at week 6	14 (47)	18 (33)	0.20
In definite or probable IFIs	5/8 (63)	5/22 (23)	0.08
In possible IFIs	9/22 (41)	13/33 (39)	0.91
Favorable response at week 12	13 (43)	16 (29)	0.19
In definite or probable IFIs	5/8 (63)	5/22 (23)	0.08
In possible IFIs	8/22 (36)	11/33 (33)	0.82
Mortality within 6 weeks			
All-cause mortality	4 (13)	14 (25)	0.19
In definite or probable IFIs	1/8 (13)	8/22 (36)	0.37
In possible IFIs	3/22 (14)	6/33 (18)	0.73
IFI-attributable mortality	3 (10)	12 (22)	0.17
In definite or probable IFIs	1/8 (13)	8/22 (36)	0.37
In possible IFIs	2/22 (9)	4/33 (12)	>0.99
Mortality within 12 weeks			
All-cause mortality	9 (30)	26 (47)	0.12
In definite or probable IFIs	1/8 (13)	11/22 (50)	0.10
In possible IFIs	8/22 (36)	15/33 (45)	0.50
IFI-attributable mortality	8 (27)	21 (38)	0.28
In definite or probable IFIs	1/8 (13)	11/22 (50)	0.10
In possible IFIs	7/22 (32)	10/33 (30)	0.91
(B) Patients Who Switched to Isavuconazole Salvage Therapy *			
**Variable**	**Monotherapy, *n* (%)** **(*n* = 27)**	**Combination Therapy, *n* (%)** **(*n* = 82)**	***p*** **-Value**
Age (years), median (range)	64 (28–80)	62 (20–86)	0.16
Sex, male	20 (74)	50 (61)	0.22
Type of IFI (definite or probable)			
*Aspergillosis*	9 (33)	22 (27)	0.52
*Mucor*	0 (0)	5 (6)	0.19
*Fusarium*	1 (4)	0 (0)	0.25
Diagnosis of IFI			0.23
Definite	0 (0)	7 (9)	
Probable	11 (41)	25 (30)	
Possible	16 (59)	50 (61)	
Invasive pulmonary infection or sinus infection	23 (85)	71 (87)	>0.99
Type of cancer			0.54
AML	16 (59)	54 (66)	
Others	11 (41)	28 (34)	
BMT-Allo	6 (22)	24 (29)	0.48
GVHD	3 (11)	11 (13)	>0.99
Neutropenia at the onset of IFI	15 (56)	56 (68)	0.23
Recovery from neutropenia during infection	6/15 (40)	23/56 (41)	0.94
Prophylaxis	18/26 (69)	53/76 (70)	0.96
Type of prophylaxis			
Polyene	0/26 (0)	1/76 (1)	>0.99
Voriconazole	4/26 (15)	19/76 (25)	0.31
Posaconazole	9/26 (35)	20/76 (26)	0.42
Isavuconazole	0/26 (0)	3/76 (4)	0.57
Echinocandins	4/26 (15)	8/76 (11)	0.50
Other	2/26 (8)	6/76 (8)	>0.99
Anti-mold prophylaxis	16/26 (62)	43/76 (57)	0.66
Primary therapy			
Polyene	10 (37)	46 (56)	0.09
Voriconazole	11 (41)	22 (27)	0.17
Posaconazole	13 (48)	53 (65)	0.13
Echinocandins	10 (37)	18 (22)	0.12
Other	0 (0)	1 (1)	>0.99
Cumulative steroids ≥ 600 mg (prednisone equivalent) during infection	6 (22)	27 (33)	0.29
ICU during infection	11 (41)	39 (48)	0.54
Mechanical ventilation during infection	4 (15)	25/81 (31)	0.10
Positive fungal culture	6 (22)	19 (23)	0.92
Species identified			0.69
*Aspergillus* spp.	4/6 (67)	9/19 (47)	
*Candida* spp.	0/6 (0)	4/19 (21)	
Others	2/6 (33)	6/19 (32)	
Co-infection	14 (52)	57 (70)	0.09
Bacterial co-infection	11 (41)	41 (50)	0.40
Viral co-infection	5 (19)	21 (26)	0.45
Favorable response at week 6	14 (52)	30 (37)	0.16
In definite or probable IFIs	4/11 (36)	11/32 (34)	>0.99
In possible IFIs	10/16 (63)	19/50 (38)	0.09
Favorable response at week 12	12 (44)	20 (24)	0.06
In definite or probable IFIs	3/11 (27)	8/32 (25)	>0.99
In possible IFIs	9/16 (56)	12/50 (24)	0.016
Mortality within 6 weeks			
All-cause mortality	6 (22)	24 (29)	0.48
In definite or probable IFIs	3/11 (27)	5/32 (16)	0.40
In possible IFIs	3/16 (19)	19/50 (38)	0.16
IFI-attributable mortality	5 (19)	21 (26)	0.45
In definite or probable IFIs	3/11 (27)	3/32 (9)	0.16
In possible IFIs	2/16 (13)	18/50 (36)	0.12
Mortality within 12 weeks			
All-cause mortality	11 (41)	45 (55)	0.20
In definite or probable IFIs	6/11 (55)	14/32 (44)	0.54
In possible IFIs	5/16 (31)	31/50 (62)	0.03
IFI-attributable mortality	8 (30)	37/81 (46)	0.14
In definite or probable IFIs	4/11 (36)	11/31 (35)	>0.99
In possible IFIs	4/16 (25)	26/50 (52)	0.06

Note: * Six patients with non-specified reasons to switch to isavuconazole salvage therapy were excluded from the analysis. ^1^ Bacterial Infection was defined as a positive bacterial culture.

**Table 3:** Comparison of different treatment combinations.

Variable	Failed Other Azole (Vori/Posa) and Switched to Isavuconazole, *n* (%) (*n* = 141) *	De Novo Isavuconazole or Failed Non-Azoles and Switched to ISA, *n* (%) (*n* = 53) **	*p*-Value
Age (years), median (range)	63 (20–90)	64 (20–91)	0.24
Sex, male	91 (65)	34 (64)	0.96
Type of IFI (definite or probable)			
*Aspergillosis*	34 (24)	20 (38)	0.06
*Mucor*	6 (4)	2 (4)	0.88
*Fusarium*	1 (1)	0 (0)	>0.99
Diagnosis of IFI			0.10
Definite	9 (6)	2 (4)	
Probable	39 (28)	23 (43)	
Possible	93 (66)	28 (53)	
Invasive pulmonary infection or sinus infection	126 (89)	47 (89)	
Type of cancer			0.11
AML	82 (58)	24 (45)	
Others	59 (42)	29 (55)	
BMT-allo	41 (29)	7 (13)	0.02
GVHD	21/140 (15)	4 (8)	0.17
Neutropenia at the onset of IFI	90 (64)	30/52 (58)	0.44
Recovery from neutropenia during infection	36/90 (40)	11/30 (37)	0.75
Cumulative steroids ≥ 600 mg (prednisone equivalent) during infection	39/140 (28)	14 (26)	0.84
Use of isavuconazole as			<0.0001
Primary therapy	36 (26)	49 (92)	
Salvage therapy	105 (74)	4 (8)	
ICU during infection	65 (46)	19 (36)	0.20
Mechanical ventilation during infection	38/140 (27)	12/52 (23)	0.57
Positive fungal culture	29 (21)	16 (30)	0.16
Species identified			0.42
*Aspergillus* spp.	12/29 (41)	10/16 (63)	
*Candida* spp.	5/29 (17)	2/16 (13)	
Others	12/29 (41)	4/16 (25)	
Co-infection	97 (69)	40 (75)	0.36
Bacterial co-infection	71 (50)	33 (62)	0.14
Viral co-infection	39 (28)	10 (19)	0.21
Favorable response at week 6	54 (38)	22 (42)	0.68
In definite or probable IFIs	17/48 (35)	8/25 (32)	0.77
In possible IFIs	37/93 (40)	14/28 (50)	0.34
Favorable response at week 12	41 (29)	20 (38)	0.25
In definite or probable IFIs	13/48 (27)	8/25 (32)	0.66
In possible IFIs	28/93 (30)	12/28 (43)	0.21
Mortality within 6 weeks			
All-cause mortality	34 (24)	14 (26)	0.74
In definite or probable IFIs	8/48 (17)	9/25 (36)	0.08
In possible IFIs	26/93 (28)	5/28 (18)	0.28
IFI-attributable mortality	29 (21)	12 (23)	0.75
In definite or probable IFIs	7/48 (15)	8/25 (32)	0.13
In possible IFIs	22/93 (24)	4/28 (14)	0.29
Mortality within 12 weeks			
All-cause mortality	69 (49)	22 (42)	0.36
In definite or probable IFIs	21/48 (44)	11/25 (44)	0.98
In possible IFIs	48/93 (52)	11/28 (38)	0.25
IFI-attributable mortality	56/140 (40)	18 (34)	0.44
In definite or probable IFIs	17/47 (36)	10/25 (40)	0.75
In possible IFIs	39/93 (42)	8/28 (29)	0.20

Note: * Six patients with non-specified reasons to switch to isavuconazole salvage therapy were excluded from the analysis. ** This group included 17 patients who had not responded to non-azoles and were switched to isavuconazole and 36 patients who had been treated de novo isavuconazole.

## Data Availability

Not applicable.
